# An exploratory study investigating the effect of targeted hyperoxemia in a randomized controlled trial in a long-term resuscitated model of combined acute subdural hematoma and hemorrhagic shock in cardiovascular healthy pigs

**DOI:** 10.3389/fimmu.2023.1123196

**Published:** 2023-04-11

**Authors:** Thomas Datzmann, Franziska Münz, Andrea Hoffmann, Elena Moehrke, Martha Binzenhöfer, Michael Gröger, Thomas Kapapa, René Mathieu, Simon Mayer, Fabian Zink, Holger Gässler, Eva-Maria Wolfschmitt, Melanie Hogg, Tamara Merz, Enrico Calzia, Peter Radermacher, David Alexander Christian Messerer

**Affiliations:** ^1^ Institute for Anesthesiological Pathophysiology and Process Engineering, Ulm University, Ulm, Germany; ^2^ Department of Anesthesiology and Intensive Care Medicine, University Hospital Ulm, Ulm, Germany; ^3^ Department of Neurosurgery, University Hospital Ulm, Ulm, Germany; ^4^ Department of Neurosurgery, German Federal Armed Forces Hospital Ulm, Ulm, Germany; ^5^ Department of Anesthesiology, Intensive Care Medicine, Emergency Medicine and Pain Therapy, German Armed Forces Hospital Ulm, Ulm, Germany; ^6^ Department of Transfusion Medicine and Hemostaseology, Friedrich-Alexander University Erlangen-Nuremberg, University Hospital Erlangen, Erlangen, Germany

**Keywords:** traumatic brain injury, multimodal brain monitoring, hemorrhagic shock, radical oxygen species, hyperoxemia

## Abstract

Severe physical injuries and associated traumatic brain injury and/or hemorrhagic shock (HS) remain leading causes of death worldwide, aggravated by accompanying extensive inflammation. Retrospective clinical data indicated an association between mild hyperoxemia and improved survival and outcome. However, corresponding prospective clinical data, including long-term resuscutation, are scarce. Therefore, the present study explored the effect of mild hyperoxemia for 24 hours in a prospective randomized controlled trial in a long-term resuscitated model of combined acute subdural hematoma (ASDH) and HS. ASDH was induced by injecting 0.1 ml × kg^−1^ autologous blood into the subdural space and HS was triggered by passive removal of blood. After 2 hours, the animals received full resuscitation, including retransfusion of the shed blood and vasopressor support. During the first 24 hours, the animals underwent targeted hyperoxemia (P_a_O_2_ = 200 – 250 mmHg) or normoxemia (P_a_O_2_ = 80 – 120 mmHg) with a total observation period of 55 hours after the initiation of ASDH and HS. Survival, cardiocirculatory stability, and demand for vasopressor support were comparable between both groups. Likewise, humoral markers of brain injury and systemic inflammation were similar. Multimodal brain monitoring, including microdialysis and partial pressure of O_2_ in brain tissue, did not show significant differences either, despite a significantly better outcome regarding the modified Glasgow Coma Scale 24 hours after shock that favors hyperoxemia. In summary, the present study reports no deleterious and few beneficial effects of mild targeted hyperoxemia in a clinically relevant model of ASDH and HS with long-term resuscitation in otherwise healthy pigs. Further beneficial effects on neurological function were probably missed due to the high mortality in both experimental groups. The present study remains exploratory due to the unavailability of an a priori power calculation resulting from the lack of necessary data.

## 1 Introduction

The presence or absence of traumatic brain injury (TBI) and/or hemorrhagic shock (HS) determines post-traumatic mortality and morbidity (1–3). TBI has been shown to be perhaps the most relevant cause of death in severely injured patients (4). In accordance, another retrospective analysis demonstrated that TBI is responsible for 58% and HS for 28% of fatal outcomes after severe injuries (5). Furthermore, TBI affects the long-term outcome after severe injuries (6–8). Current treatment regimens recommend avoiding cerebral hypoxemia, which is largely affected by maintaining a cerebral perfusion pressure of 60 – 70 mmHg and by preventing systemic hypoxemia (9, 10). Based on simple physiological considerations and the corresponding evidence, systemic hyperoxemia may be useful to avoid hypoxemia and related detrimental inflammation in regions with reduced availability of oxygen transport molecules (for example, due to compromised local circulation caused by decreased CPP and/or anemia due to HS) (11–14). However, hyperoxemia is associated with several negative effects such as increased generation of reactive oxygen species (ROS) and is therefore controversially discussed (15–17). Nevertheless, due to its properties as a vasoconstrictor, oxygen may ameliorate brain injury by improving cranial perfusion without reducing tissue oxygenation (15, 17).

Current concepts suggest that severe hyperoxemia (arterial blood oxygen tension (PaO_2_) > 300 mmHg (40 kPa)) should be avoided (15, 17, 18). However, a potential ‘sweet spot’ of mild hyperoxemia remains unknown. The retrospective data coincided with mild hyperoxemia (PaO_2_ > 150 and < 200 mmHg) with improved survival and outcome, respectively, within the first 24 hrs (19, 20). In accordance, mild hyperoxemia (PaO_2_ of 150 – 250 mmHg during the first 24 hrs of intensive care unit stay) coincided with improved functional outcome and better survival (21). In accordance, a recent meta-analysis reported that normobaric hyperoxia may improve metabolic alterations after acute brain injury (22).

Taken together, there is an imminent clinical need to improve survival and outcome after combined HS and TBI. To our knowledge, there are no long-term prospective experimental or clinical trials investigating the amount of hyperoxemia and the suitable period of hyperoxemia after TBI and/or HS. In this context, we recently demonstrated in an explorative study, that mild hyperoxemia is not deleterious, but shows a tendency toward beneficial effects in a porcine model of combined TBI and HS in pigs challenged with preexisting atherosclerosis (23).

Moreover, we had previously shown that the efficacy of therapeutic interventions in shock states depends not only on the severity of the shock (24, 25) but also on the presence or absence of underlying chronic cardiovascular comorbidity (26–29). Therefore, the present prospective randomized controlled experimental study investigated the effects of targeted moderate hyperoxemia in cardiovascular healthy swine. As in our previous investigation (23), the main outcome parameters were whether this approach (i) ameliorates brain tissue oxygenation, (ii) improves neurologic function, and (iii) is safe with respect to parameters of oxidative stress.

## 2 Materials and methods

### 2.1 Animals

Ethical approval was obtained from the local Animal Care Committee of Ulm University and the Federal Authorities (Tuebingen, Germany) for Animal Research (#1316). All experiments were carried out in full compliance with the National Institute of Health Guidelines on the Use of Laboratory Animals and the European Union ‘Directive 2010/63/EU on the protection of animals used for scientific purposes’. Fourteen adult pigs (body weight: 75 kg (73,76), age: 16 months (15; 18) of both sexes (4 females and 10 castrated males) were purchased from the Hôpital Lariboisière, Paris, France. The pigs were of the Bretoncelles-Meishan-Willebrand strain, which presents with a reduced activity of the von Willebrand factor (vWF), thus mimicking the human coagulation system (24, 25, 30, 31), in contrast to the hypercoagulatory state in domestic swine strains (32). The animals were sheltered at Oberberghof, Ulm, Germany, until further use with an acclimatization period of at least two weeks. The animals were kept at a cycle of 12/12 hrs light/darkness and were monitored at least once daily. The housing temperature was set to 21 – 22°C with a humidity of 50 – 60%.

To our knowledge, as in a related study (23) that employs pigs with preexisting coronary artery disease to acute subdural hematoma (ASDH) and HS with or without targeted hyperoxemia for 24 hrs, no available data from previous studies are available, which would allow an a priori sample size calculation. Therefore, and because retrospective clinical data and pathophysiological considerations suggest a usefulness of moderate hyperoxemia after traumatic brain injury and hemorrhage, as outlined in the introduction, sample size calculation was difficult. To address this important research gap, this exploratory study was conducted with a limited animal size (seven per group) to generate and disseminate important data on whether targeted hyperoxemia is detrimental or beneficial and to facilitate the calculation of the sample size for subsequent trials.

### 2.2 Anesthesia and instrumentation

Anesthesia and surgical instrumentation were identical to the procedures previously described in swine with coronary artery disease (23, 30, 31, 33). The animals had free access to water and obtained a nutritional solution (Fresubin, Fresenius Kabi, Bad Homburg, Germany) in the last 12 hrs before the experiment. Prior to instrumentation, the animals were sedated by intramuscular injection of azaperone (5 mg × kg^−1^ (milligram per kilogram of body weight)) and midazolam (1 – 2 mg × kg^−1^). Next, an intravenous catheter was established in an auricular vein. Anesthesia was induced by intravenous injection of propofol (1 – 2 mg × kg^−1^) and ketamine (1 mg × kg^−1^). Subsequently, the pigs were endotracheally intubated and mechanically ventilated (ventilator settings: tidal volume 8 ml × kg^−1^, respiratory rate 8 – 12 breaths per min adapted to achieve an arterial PCO_2_ (P_a_CO_2_) of 35 – 40 mmHg, inspiratory/expiratory (I/E) ratio of 1:1.5, fraction of inspiratory oxygen (F_I_O_2_) of 0.3, positive end-expiratory pressure (PEEP) 10 cm H_2_O to reduce atelectasis formation). Anesthesia was maintained by continuous infusion of propofol (10 mg × kg^−1^ × h^−1^) and remifentanil (initial bolus: 5 mg × kg^−1^, followed by 15 – 20 µg × kg^−1^ × h^−1^). To maintain fluid balance, a balanced electrolyte solution (10 ml × kg^−1^ × h^−1^, Jonosteril 1/1, Fresenius Kabi) was infused. During instrumentation, hydroxyethyl starch 6% 130/0.42 (Vitafusal, Serumwerk, Bernburg) was infused with a maximum dose of 30 ml × kg^−1^ to stabilize circulation if necessary. After surgical exposure, a 4-lumen venous catheter (7 Fr, Teleflex, Reading, USA) was placed in the right iliac vein to measure the central venous pressure, to return the shed blood, and to administer the required medication. The resulting central venous pressure should be interpreted with caution, because the tip of the catheter was located in the inferior vena cava and due to the influence of the applied PEEP. In addition, for continuous measurement of cardiac output, pulse pressure, and stroke volume variation, a 5-F PiCCO catheter (PULSION Medical Systems, Munich, Germany) was inserted into the right femoral artery. Moreover, a 10-F sheath (Super Arrow-Flex Percutaneous Sheath Introducer Set, Teleflex) was placed in the left femoral artery for the induction of the hemorrhage shock by passive removal of blood and for blood sampling as described below. A midline minilaparotomy was performed to insert a catheter into the urinary bladder. Subsequently, the pigs were put in a prone position for the neurosurgical instrumentation consisting of a craniotomy over both parietal cortices. For the induction of ASDH later, the dura was opened and a ventricular catheter (9F, Neuromedex, Hamburg, Germany) was inserted approximately 5 mm into the subdural space. The catheter was placed alternating between each experiment in proximity to either the left or right hemisphere. Subsequently, microdialysis catheters (see below) and multimodal brain monitoring probes (Neurovent-PTO, Raumedic AG, Helmbrechts, Germany) were inserted approximately 10 – 15 mm into the parenchyma of both hemispheres. These multimodal brain monitoring probes were used for the measurement of intracranial pressure (ICP) and partial pressure of O_2_ in brain tissue (P_bt_O_2_). After equilibration of both catheters and stabilization of P_bt_O_2_, recording was started according to the manufacturer’s instructions. In the end, bone wax was used to close the burr holes as well as to fix the microdialysis catheter and the multimodal brain monitoring probes. Neurosurgical instrumentation of both hemispheres was conducted to eliminate the need for sham experiments (3R principles (34)), the hemisphere without ASDH was used as a control for the hemisphere with ASDH. Body temperature was assessed by a rectal probe. The animals were kept at a temperature of 37 – 38°C. After the initiation of resuscitation, the temperature was controlled to maintain brain normothermia. If the brain temperature reached ≥ 39°C, the pigs received external cooling by ice-cold water-filled bags.

### 2.3 Experimental approach

**Figure 1:**
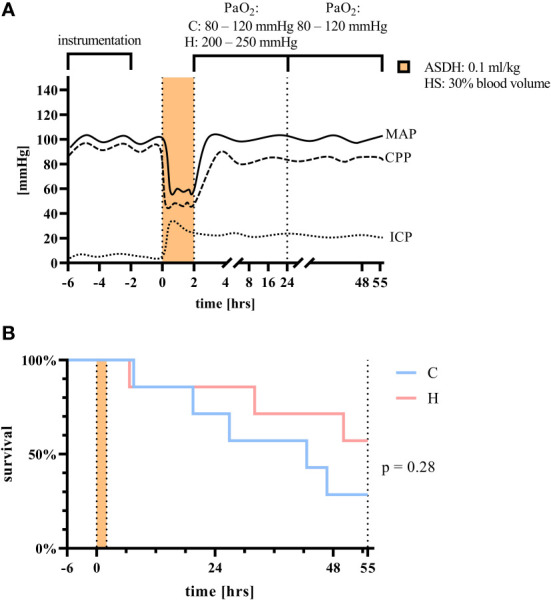
**(A)** Experimental setup and **(B)** survival analysis of animals treated with normoxemia as control (C, blue) or hyperoxemia (H, red). The dotted, dashed, or filled line refers to schematic trends in intracranial pressure (ICP), cranial perfusion pressure (CPP), and mean arterial pressure (MAP), respectively. Orange indicates the phase of combined hemorrhagic shock (HS) and acute subdural hematoma (ASDH). Mantel-Cox test, n = 7 per group.

**Figure 1A** summarizes the experimental protocol, which was similar to a recently published study in pigs with preexisting cardiovascular disease (23). The protocol consisted of an instrumentation period (4 hrs), a rest period (2 hrs), a period of combined ASDH and HS (2 hrs), a resuscitation period (24 hrs) that included the intervention of either applying normoxemia (target P_a_O_2_ = 80 – 120 mmHg) or hyperoxemia (target P_a_O_2_ = 200 – 250 mmHg), and a further period of resuscitation with normoxemia (24 hrs + 7 hrs). The total experimental period after the induction of ASDH and HS was 55 hrs.

To mimic the typical clinical situation where resuscitation procedures are initiated with a certain delay after trauma and hemorrhage, the fluid infusion rate (Lactated Ringer’s solution) was reduced to 100 ml × h^−1^, the ventilator settings were changed to a tidal volume of 8 ml × kg^−1^, PEEP 0 cmH_2_O, I/E ratio 1:2, F_I_O_2_ 0.21. To initiate ASDH, 0.1 ml × kg^−1^ of autologous blood was injected using an automated syringe pump for 15 min through the subdural catheter. This approach was chosen based on the rationale that in previous studies on porcine ASDH, the injection of a blood volume approximating 10% of the intracranial volume represents the threshold for supratentorial volume tolerance (35, 36). Following the induction of ASDH, HS was initiated by passive removal of blood for 30 min using the large bore arterial catheter targeting 30% of the calculated blood volume (23, 26). The removed blood was stored at 4 – 8°C in acid-citrate-dextrose solution until retransfusion. Blood removal was decelerated or interrupted as necessary to maintain cerebral perfusion pressure (CPP, difference between MAP and ICP)) ≥ 50 mmHg. This regimen was chosen to prevent irreversible brain damage based on previous experiences. After two hours of combined ASDH (15 min) and HS (105 min), resuscitation was initiated. Resuscitation included retransfusion of the shed blood within 30 min, fluid resuscitation (20 ml × kg^−1^ × h^−1^ Ionosteril, reduced to 10 ml × kg^−1^ × h^−1^ if central venous pressure > 16 mmHg) as well as by continuous i.v. noradrenaline titrated to the MAP at pre-shock levels (± 10%) and CPP at baseline levels and at least > 60 mmHg (the latter as referred to in current guidelines (9)).

Upon initiation of resuscitation, the baseline ventilator settings were set to tidal volume 8 ml × kg^−1^, respiratory rate 8 – 12 breaths per min to maintain PaCO_2_ of 35 – 40 mmHg, I/E ratio of 1:1.5, and PEEP 10 cm H_2_O. During the first 24 hrs of treatment, the animals were randomly assigned to either receive targeted hyperoxemia (P_a_O_2_ = 200 – 250 mmHg) or normoxemia (PaO_2_ = 80 – 120 mmHg). This PaO_2_ target window was chosen because it had coincided with a lower lethality and improved neurological outcome in patients with a severe TBI interval (21). After 24 hrs, both groups were treated with normoxemia until the end of the experiments. Following further deepening of the anesthesia, the pigs were sacrificed by injection of potassium chloride. Animals were euthanized before the end of the predetermined 55-hrs monitoring and treatment period in case of CPP < 60 mmHg despite the maximum dose of vasopressors (limited to a heart rate of 160 per min to prevent myocardial injury induced by tachycardia) or acute anuric kidney failure with consecutive hyperkalemia (blood potassium > 6 mmol × l^−1^) and cardiac arrhythmia.

### 2.4 Measurements

Sampling of most parameters was carried out 30 min before the start of ASDH and HS (baseline, referred to as ‘pre’ in figures and tables), at the end of the 2 hrs ASHD and HS period (‘post’), after 24 hrs and after 48 hrs via the arterial catheter inserted in the femoral artery. Arterial sampling was chosen to prevent potential interferences with the administration of drugs and infusions, to reduce the need for additional venous instrumentation, and to ensure rapid sampling. In addition, this sampling method can be considered to analyze the systemic levels of the analytes, which are distributed to all organs (and thus potentially affecting them) and to result in well-mixed samples affected by all organs and tissues compared to a possible sampling from the iliac vein (the latter in which analytes from the cerebral circulation would rather be metabolized or otherwise influenced, for example, in their concentration and biochemical identity).

Hemodynamics, gas exchange (calorimetric O_2_ uptake and CO_2_ production), arterial blood gas tensions, acid-base status, glucose, lactate, creatinine, neutrophil gelatinase-associated lipocalin (NGAL), 8-isoprostane, bilirubin, and troponin were determined as previously described (29, 30, 37). In brief, blood gas analysis, glucose, and lactate levels were measured using a standard blood gas analyzer (ABL 800 Flex, Radiometer GmbH, Krefeld, Germany). 8-isoprostane (#516351, Cayman Chemical, Ann Arbor, USA), and troponin (#2010-4-HSP, Life Diagnostics, West Chester, USA), tumor necrosis factor (TNF, #PTA00, R&D Systems, Minneapolis, USA), interleukin 6 (IL6, #P6000B, R&D Systems), interleukin 10 (IL10, #P1000, R&D Systems), enolase (porcine neuron specific enolase, #E07N0025, BlueGene Biotech, Shanghai, China), MAP 2 (porcine microtubule-associated protein 2, #E07M0226, BlueGene Biotech), GFAP (porcine glial fibrillary acidic protein, #E07G0157, BlueGene Biotech), and S100B (porcine S100 Calcium Binding Protein B) were determined as recommended by the manufacturer.

The neurological function was evaluated using a swine-adapted modified Glasgow Coma Scale (MGCS) as described in detail previously (31). To this end, the depth of anesthesia was reduced until appropriate spontaneous breathing was resumed at baseline as well as after 24 hrs and 48 hrs of treatment.

Intracerebral tissue metabolites (glutamate, lactate, pyruvate, and glucose) were determined using an automated microdialysis system (CMA 600 Microdialysis Analyzer, CMA/Microdialysis AB, Kista, Sweden). Microdialysis catheters (70 microdialysis bolt catheter, M Dialysis AB) were implanted bilaterally after dura perforation, lowered to a depth of 10 – 15 mm, and perfused (perfusion fluid, CMA/Microdialysis AB) by a microdialysis pump (CMA/102 microdialysis pump, CMA/Microdialysis AB). After calibration according to the manufacturer’s specifications, microdialysate samples were collected in microvials (MDialysis AB) over 2 hrs (i.e., from 1 hrs before until 1 hrs after each measurement time point) and analyzed immediately after collection. At the baseline measurement time point, an additional first hour of dialysate was collected but discarded to facilitate equilibration of the dialysis process.

Superoxide anion radical concentrations (O_2_^·−^) in arterial blood were measured as previously described (23, 38). Briefly, blood and the O_2_^·−^-specific spin probe 1-hydroxy-3-methoxycarbonyl-2,2,5,5-tetramethylpyrrolidine (CMH, 200 µM final concentration) solved in Krebs-Hepes-Buffer containing the metal chelators deferoxamine-methanesulfonate (DF) and diethyldithiocarbamic-acid (DETC) (all from Noxygen, Elzach, Germany) were mixed. After transfer to a glass capillary tube and incubation at 37°C, the sample was analyzed with an EMXnano ESR spectrometer (Bruker, Billerica, USA) equipped with a temperature controller (BIO-III R, Noxygen). For each sample, three scans were averaged. When the amplitude of the sample spectrum was compared with that of a standard dilution series of the stable radical 1-hydroxy-3-methoxycarbonyl-2,2,5,5-tetramethylpyrrolidine (CP^·^) after subtraction of the blank solution, the resulting values allowed the determination of O_2_^·−^-concentrations.

### 2.5 Data analysis

Survival was analyzed using a Kaplan-Meier graph followed by Log-rank (Mantel-Cox) test. Experimental data was considered to be nonparametric. The comparison of treatment and vehicle group was carried out by means of the Mann-Whitney U test. Data is graphed in boxplots with median as well as 25^th^ and 75^th^ percentile. Whiskers indicate upper and lower extremes, respectively. In the manuscript, the data is reported as median in conjunction with the 25^th^ percentile and the 75^th^ percentile. Due to the sample size and the exploratory character of the study, no general exclusion of potential outliers was performed. Single data points, which were highly suspect of being an outlier, were identified by manual screening or according to individual experimental records (for example, a defect in a measurement or sampling device). Statistical analysis was performed with GraphPad Prism9 (GraphPad Software, Inc., San Diego, California, USA).

## 3 Results

### 3.1 General group characteristics and survival

The group allocation resulted in a similar distribution with respect to the weight of the animals (normoxemia: 75 kg (72, 76) vs. hyperoxemia: 74 kg (74, 79)), side of the ASDH (normoxemia: 4 left/3 right vs. hyperoxemia: 3 left/4 right), sex (normoxemia: 4/7 male castrated vs hyperoxemia. 6/7 male castrated), and removed blood volume in percent to the total calculated blood volume to achieve HS as described above (normoxemia: 14.5% (13.0%; 22.4%) vs. hyperoxemia: 18.4% (16.7%; 24.9%).

Survival did not differ significantly between the two groups (**Figure 1B**, p = 0.28, Mantel-Cox test). One experiment per group had to be terminated prematurely due to kidney failure leading to hyperkalemia and subsequent arrhythmias. All other experiments that had to be ended before the total observation period of 55 hrs were terminated due to a drop in CPP as described in the methods section.

### 3.2 Parameters of hemodynamics, gas exchange, acid-base status, and humoral markers of inflammation and brain injury

**Tables 1**, **2** summarize the parameters with a focus on cardiorespiratory functions, kidney function, and complete blood count. As expected, due to the intervention, there were significant differences in P_a_O_2_ levels and P_a_O_2_/F_I_O_2_ ratio after 24 hrs. All other assessed parameters did not show significant differences. Interestingly, this also included the presence of superoxide anions in whole blood, which did not differ significantly between the normoxemia group and the hyperoxemia group during the observation period (**Table 1**). In addition, the noradrenaline infusion rates needed to achieve hemodynamic targets were similar (normoxemia: 0.86 µg × kg^−1^ × min^−1^ (0.58; 0.97) vs. hyperoxemia: 0.78 µg × kg^−1^ × min^−1^ (0.38; 1.24), p = 0.94, Mann-Whitney test).

**Table 1:** Organ function parameters for the heart, the lungs, and the kidneys before (pre) and after 2 hrs of hemorrhagic shock and acute subdural hematoma (post) as well as 24 hrs and 48 hrs after resuscitation.

Parameter	Group	pre	post	24 hrs	48 hrs
Body Temperature (°C)	C	35.8 (35.0; 35.8)	35.3 (35.0; 36.3)	37.1 (36.9; 38.1)	38.0 (37.5; 38.1)
	H	35.0 (34.8; 36.0)	34.8 (34.2; 36.1)	37.9 (37.2; 38.3)	37.8 (37.6; 38.2)
Heart Rate (beats × min^−1^)	C	64 (56, 71)	130 (123, 134)	59 (52; 65)	96 (54; 150)
	H	63 (49; 71)	127 (107; 133)	55 (53; 60)	73 (64; 110)
Stroke Volume (ml)	C	101 (90; 117)	34 (29; 48)	100 (76; 114)	95 (70; 99)
	H	114 (102; 133)	34 (32; 42)	95 (79; 107)	88 (57; 103)
Stroke Volume Variation (%)	C	8 (6; 11)	12 (10; 14)	12 (10; 17)	5 (4; 6)
	H	10 (5; 12)	12 (10; 13)	8 (5; 15)	7 (5; 14)
Cardiac Output (l × min^−1^)	C	6.4 (6.1; 7.9)	4.5 (3.9; 6.0)	6.2 (5.1; 8.1)	9.5 (5.7; 10.3)
	H	6.4 (4.8; 7.0)	4.1 (2.7; 4.5)	5.3 (4.6; 6.3)	6.4 (4.4; 9.6)
MAP (mmHg)	C	98 (90; 105)	63 (61; 72)	119 (117; 144)	110 (101; 155)
	H	99 (91; 112)	61 (57; 67)	131 (125; 136)	115 (109; 124)
Central Venous Pressure^a^ (mmHg)	C	8 (7; 10)	4 (2; 5)	13 (10; 13)	12 (12; 12)
	H	8 (6; 9)	5 (3; 6)	12 (11; 14)	11 (9; 14)
Urine Output (ml^−1^ × h^−1^ × kg^−1^)	C	10.7 (5.8; 12.6)	not measured	5.7 (1.3; 6.4)	9.4 (1.6; 9.8)
	H	10.9 (8.3; 11.5)	not measured	4.7 (3.8; 6.2)	5.2 (3.0; 6.0)
Positive end-expiratory pressure (cmH_2_O)	C	0 (0; 0)	0 (0; 0)	10 (10; 10)	10 (10; 10)
	H	0 (0; 0)	0 (0; 0)	10 (10; 10)	10 (10; 10)
Respiratory Minute Volume (l × min^−1^)	C	4.5 (4.2; 5.5)	4.5 (3.9; 5.2)	5.9 (5.7; 29.4)	6.0 (5.3; 6.8)
	H	4.8 (4.5; 5.1)	4.8 (4.5; 5.3)	6.7 (6.4; 7.3)	7.0 (6.8; 8.8)^b^
Arterial PO_2_ (mmHg)	C	71 (65; 87)	88 (84; 104)	85 (80; 99)	90 (79; 107)
	H	76 (65; 92)	95 (77; 104)	233 (216; 243)**	89 (87; 95)
PaO_2_/F_I_O_2_ ratio (mmHg)	C	338 (311; 413)	417 (402; 495)	397 (371; 415)	391 (317; 428)
	H	361 (311; 437)	453 (365; 495)	491 (479; 540)**	387 (358; 409)
Arterial PCO_2_ (mmHg)	C	38.1 (37.1; 42.5)	38.7 (33.8; 45.7)	40.7 (37.9; 41.2)	38.8 (38.4; 44.1)
	H	36.0 (35.0; 41.2)	41.6 (36.7; 43.5)	39.0 (34.1; 41.5)	38.4 (35.7; 43.8)
Oxygen Consumption (ml × min^−1^)	C	185 (176; 218)	166 (153; 185)	215 (190; 265)	235 (207; 285)
	H	179 (160; 194)	144 (130; 189)	194 (135; 237)	233 (209; 246)
Carbon Dioxide Generation (ml × min^−1^)	C	136 (120; 163)	136 (120; 155)	175 (130; 203)	202 (187; 243)
	H	138 (121; 146)	119 (105; 154)	190 (167; 208)	211 (183; 236)
Troponin (ng × ml^−1^)	C	0.03 (0.02; 0.19)	0.07 (0.02; 0.16)	1.30 (0.81; 4.04)	0.60 (0.16; 0.78)
	H	0.02 (0.02; 0.02)	0.02 (0.02; 0.02)	0.41 (0.04; 1.71)	0.06 (0.01; 1.90)
NGAL (ng × ml^−1^)	C	303 (259; 356)	379 (334; 633)	726 (507; 1 865)	748 (577; 2 079)
	H	311 (270; 383)	438 (392; 570)	523 (507; 1 163)	825 (783; 2 167)
Superoxide Anion (µm × l^−1^)	C	5.7 (5.6; 6.1)	5.6 (4.7; 5.9)	6.5 (5.4; 6.8)	5.2 (4.5; 6.0)
	H	6.3 (5.2; 6.4)	5.2 (4.9; 5.4)	6.4 (5.2; 7.0)	6.2 (5.3; 6.6)

n = 7/7 before shock, 7/7 after shock, 5/6 at 24 hrs, and 3/5 at 48 hrs per group for normoxemia (blue, control group, C) and targeted hyperoxemia (red, H), respectively. For the central venous pressure^a^, see method section for further details. Values are reported as median and interquartile range. p-values are reported when below 0.1, ^b^ denotes p = 0.07, ** = p < 0.01, Mann-Whitney U test.

**Table 2:** Glucose, lactate, pH, base excess, and complete blood count before (pre) and after 2 hrs of hemorrhagic shock and acute subdural hematoma (post) as well as 24 hrs and 48 hrs after resuscitation.

Parameter	Group	pre	post	24 hrs	48 hrs
Arterial Glucose (mmol × l^−1^)	C	4.6 (3.8; 4.9)	4.2 (3.8; 4.8)	4.4 (3.6; 4.6)	3.7 (3.2; 4.2)
	H	4.1 (3.9; 4.3)	3.9 (3.6; 4.4)	4.2 (3.8; 5.3)	4.4 (3.9; 5.0)
Lactate (mmol × l^−1^)	C	2.0 (1.6; 2.2)	2.6 (2.1; 3.9)	1.0 (0.8; 1.5)	1.0 (0.5; 1.0)
	H	1.8 (1.7; 2.4)	3.1 (2.9; 3.9)	1.0 (0.5; 1.2)	0.7 (0.6; 0.8)
pH	C	7.50 (7.44; 7.54)	7.51 (7.41; 7.54)	7.45 (7.41; 7.51)	7.50 (7.34; 7.55)
	H	7.50 (7.45; 7.52)	7.47 (7.45; 7.51)	7.51 (7.50; 7.52)	7.50 (7.41; 7.55)
Base Excess (mmol × l^−1^)	C	4.7 (4.3; 6.9)	6.5 (4.1; 7.6)	4.3 (1.7; 6.4)	6.3 (–1.7; 10.1)
	H	5.1 (4.0; 6.3)	5.8 (4.6; 6.0)	6.1 (4.1; 10.0)	6.6 (1.7; 9.5)
Sodium (mmol × l^−1^)	C	143 (142; 144)	142 (141; 145)	146 (140; 150)	140 (138; 147)
	H	144 (143; 145)	143 (142; 144)	148 (146; 151)	144 (143; 148)
Potassium (mmol × l^−1^)	C	3.2 (3.1; 3.4)	3.6 (3.4; 3.7)	3.7 (3.2; 5.5)	3.3 (2.7; 4.1)
	H	3.1 (3.0; 3.2)	3.5 (3.4; 3.6)	3.4 (2.8; 3.9)	3.1 (2.9; 3.8)
Hemoglobin (g × dl^−1^)	C	8.4 (8.2; 8.7)	7.9 (7.8; 8.8)	11.0 (9.5; 11.2)	9.4 (6.8; 11.5)
	H	8.4 (7.9; 8.7)	7.4 (7.0; 8.4)	9.9 (8.8; 10.1)	8.6 (7.8; 9.8)
Erythrocytes (× 10^15^ × l^−1^)	C	4.4 (4.2; 4.5)	4.1 (4.0; 4.4)	5.6 (4.9; 6.1)	5.1 (3.6; 6.1)
	H	4.6 (4.2; 4.9)	4.1 (3.8; 4.4)	5.2 (4.6; 5.4)	4.5 (4.2; 5.0)
Thrombocytes (× 10^9^ × l^−1^)	C	144 (137; 172)	151 (143; 193)	46 (29; 92)	58 (28; 114)
	H	170 (127; 183)	181 (165; 205)	104 (65; 133)	58 (39; 93)
Leukocytes (× 10^12^ × l^−1^)	C	4.4 (3.7; 6.0)	4.5 (3.1; 5.9)	14.9 (9.1; 20.6)	11.3 (8.8; 25.2)
	H	4.3 (3.6; 7.1)	4.3 (3.4; 7.4)	16.4 (10.4; 19.5)	13.7 (8.4; 18.4)

n = 7/7 before shock, 7/7 after shock, 5/6 at 24 hrs, and 3/5 at 48 hrs per group for normoxemia (blue, control group, C) and targeted hyperoxemia (red, H), respectively. Values are reported as median and interquartile range. p-values are reported when below 0.1, Mann-Whitney U test.

**Figure 2:**
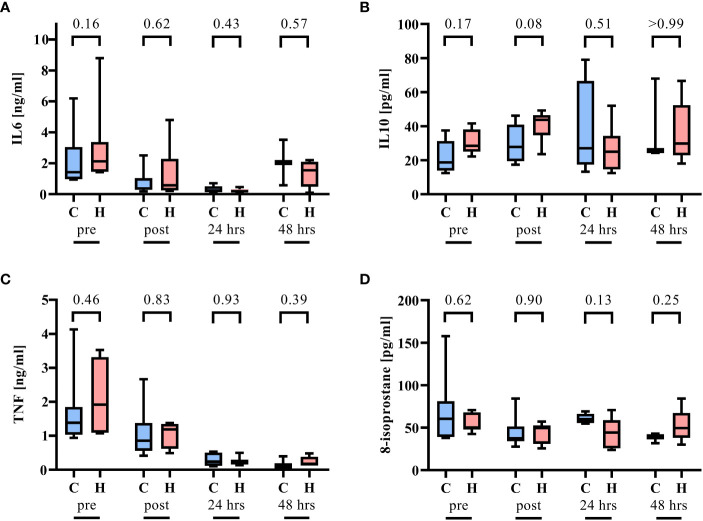
Inflammation parameters before (pre) and after 2 hrs of hemorrhagic shock and acute subdural hematoma (post) as well as 24 hrs and 48 hrs after resuscitation. n = 7/7 before shock, 7/7 after shock, 5/6 at 24 hrs, and 3/5 at 48 hrs per group for normoxemia (blue, control group, C) and targeted hyperoxemia (red, H), respectively. **(A)** Interleukin 6 (IL6), **(B)** interleukin 10 (IL10), **(C)** tumor necrosis factor, and **(D)** 8-isoprostane. Mann-Whitney U test. The box plots report the median, interquartile range, minimum, and maximum.

**Figure 3:**
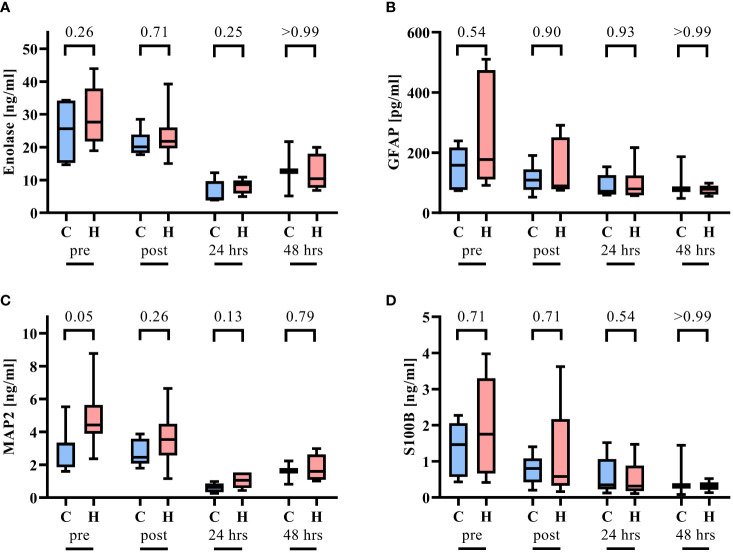
Parameters of neuronal injury before (pre) and after 2 hrs of hemorrhagic shock and acute subdural hematoma (post) as well as 24 hrs and 48 hrs after resuscitation n = 7/7 before shock, 7/7 after shock, 5/6 at 24 hrs, and 3/5 at 48 hrs per group for normoxemia (blue, control group, C) and targeted hyperoxemia (red, H), respectively. **(A)** Enolase, **(B)** porcine glial fibrillary acidic protein (GFAP), **(C)** porcine microtubule-associated protein 2 (MAP2), and **(D)** porcine S100 Calcium Binding Protein B (S100b). For MAP2 before shock and Enolase 24 hrs after resuscitation in the normoxemia group and GFAP post HS and ASDH in the hyperoxemia group, the 25^th^ percentile and the median are similar and therefore could not be graphed separately. Mann-Whitney U test. The box plots report the median, interquartile range, minimum, and maximum.

The levels of IL6, IL10, TNF, and 8-isoprostane were similar during the experiments and compared between both groups (**Figure 2**). Similarly, there were no significant differences in the analysis of enolase, GFAP, MAP2 and S100B between both groups (**Figure 3**).

### 3.3 Brain monitoring and MGCS

**Figure 4:**
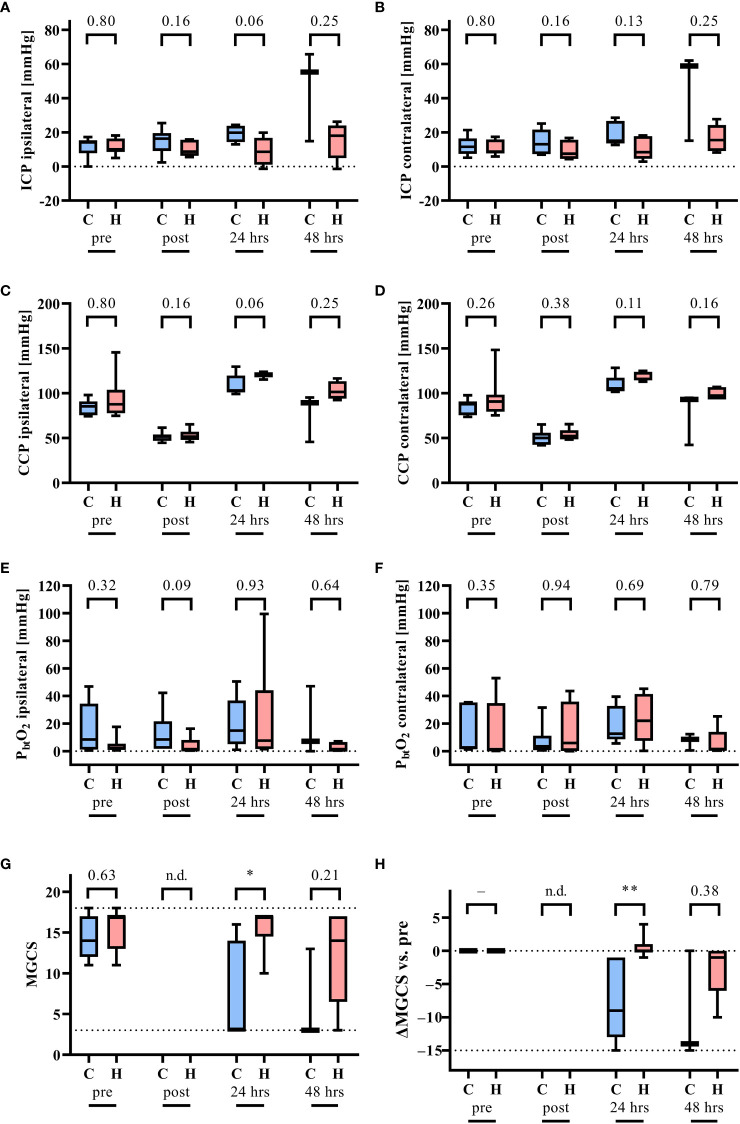
Monitoring of intracranial pressure (ICP), the cerebral perfusion pressure (CCP) and the partial pressure of O_2_ in brain tissue (P_bt_O_2_) at the site of ASDH (ipsilateral) and the opposing hemisphere (contralateral). Measurements were recorded before (pre) and after 2 hrs of hemorrhagic shock and acute subdural hematoma (post) as well as 24 hrs and 48 hrs after resuscitation n = 7/7 before shock, 7/7 after shock, 5/6 at 24 hrs, and 3/5 at 48 hrs per group for normoxemia (blue, control group, C) and targeted hyperoxemia (red, H), respectively. **(A)** ICP ipsilateral, **(B)** ICP contralateral, **(C)** CCP ipsilateral, **(D)** CCP contralateral, **(E)** P_bt_O_2_ ipsilateral, **(F)** P_bt_O_2_ contralateral, **(G)** modified Glasgow Coma Scale (MGCS), and **(H)** the difference of the MGCS in comparison to before shock. The dotted lines indicate the minimal and maximal possible values as for MGCS. Mann-Whitney U test. * = p < 0.05, ** = p < 0.01. n.d. = not determined. The box plots report the median, interquartile range, minimum, and maximum.

ICP and (P_bt_O_2_) did not differ in the ipsilateral or contralateral brain hemisphere between the groups (**Figure 4**). Interestingly, systemic hyperoxemia, as confirmed by blood gas analysis, did not have a significant impact on P_bt_O_2_. Likewise, the analysis of glutamate, glucose, pyruvate, and lactate was comparable (**Supplemental Figure 1**). The MGCS values were similar at baseline and declined after 24 hrs and 48 hrs (**Figure 4**). For absolute and for changes in MGCS compared to the baseline, there was a significant improvement in MGCS in the hyperoxemia group after 24 hrs.

## 4 Discussion

The main result of the present study was that in a long-term resuscitated model of combined ASDH and HS that mild hyperoxemia did not show deleterious effects but a weak trend towards beneficial effects regarding survival. Further major findings include no increased generation of systemic ROS as well as an improved MGCS 24 hrs after shock.

The findings are consistent with previous retrospective studies that indicate beneficial effects of mild hyperoxemia in the first 24 hrs after the incident in patients with severe injuries and/or TBI (19–21). Hyperoxemia might exert in principal positive and negative effects (15, 39). The total arterial oxygen content is mainly determined by the amount of oxygen bound to hemoglobin and to a small extent only to the proportion of oxygen, which is physically dissolved [see **Figure 4** (39)]. Therefore, increasing the partial oxygen P_a_O_2_ has only little impact on the total arterial oxygen content. However, according to Fick’s law, the P_a_O_2_ represents the upstream oxygen partial pressure of the diffusion gradient determining the diffusion distance of oxygen within the tissues [see **Figure 1B** (40)]. Although it is tempting to speculate that even these small increases in arterial oxygen content might slightly improve local oxygen supply (Letzte Wiese/Last meadow concept) (41), these effects are difficult to measure in terms of tissue oxygenation, but might contribute to the observed improved neurological outcome. However, hyperoxemia has a multitude of systemic effects, including pulmonic vasodilation and thereby reduction of right ventricular load, but otherwise systemic and cerebral vasoconstriction, which in turn might ameliorate cerebral perfusion and relieve intracranial pressure (11, 12, 14, 39). Furthermore, hyperoxemia might induce several changes in metabolism. For example, this might affect the respiratory quotient (42) indicating enhanced carbohydrate metabolism. This effect has been observed during resuscitated fecal peritonits-induced porcine septic shock (43) and is likely associated with more ATP production per mole of oxygen used (44), i.e., improved yield of cellular energy metabolism (45). The consequences of these systemic effects in the setting of ASDH and HS in dependence of hyperoxemia warrant further investigation. It is noteworthy that, albeit not significantly different, the two survival curves split shortly after the end of the hyperoxemia period. On the one hand, the present study reports, in contrast to our previous study in pigs with atherosclerosis (23), no effect on P_bt_O_2_ during mild hyperoxemia. Of note, atherosclerosis is associated with chronic inflammation, increased oxidative stress, and decreased cerebral blood flow (46, 47). On the other hand, this finding is consistent with a study by Hawryluk et al. (48) and might be explained by the positioning of the P_bt_O_2_ probes. In agreement with our previous work (23), transient targeted hyperoxemia affected neither the concentration of superoxide anion (a marker of ROS formation) nor isoprostane (a marker of lipid peroxidation). In this regard, previous experimental and clinical data yielded ambiguous results on oxidative stress after TBI, including increased (49), unchanged (50, 51), or decreased (52) markers of radical damage.

This study has several strengths and limitations. An a priori power analysis was impossible to calculate (e.g., the sample size regarding outcomes such as survival and MGCS) due to the lack of available data. Moreover, a potential beneficial impact of mild hyperoxemia on MGCS might have been missed due to the lower survival in the normoxemia group. Additionally, the MGCS was not blinded, thereby not certainly excluding a potential bias. However, the significantly improved MGCS is supported by several other trends such as decreased ICP and potential improved survival. Nevertheless, this study generated important data for future trial designs. In addition, several interesting observations were observed, e.g., decreased platelet count after 24 hrs in the hyperoxemia group (**Table 2**), which, however, did not reach statistical significance. These findings, which are in accordance to platelet trapping observed in a murine model of hyperoxia (53), are currently difficult to interpret due to the exploratory nature of this study, but provide a rationale for further exploration in a follow-up study. In this context, it is noteworthy that the survival curve split after approximately 24 hrs but several parameters realigned after 48 hrs. Although this circumstance should not be overinterpreted, one might speculate that during hyperoxemia, animals appear to be less affected by previous HS and ASDH.

This clinically relevant model of combined HS and ASDH with delayed and long-term resuscitation did not find strong detrimental effects of mild hyperoxemia and therefore corroborates existing retrospective clinical data (19–21). In this context, it should be noted that the present study induced brain injury by acute subdural hematoma, which mimics a detrimental mass effect but not axonal injury, for example, due to direct kinetic impact. Although this approach facilitates standardization, the present data set should be analyzed with regard to the impact of mass effects due to traumatic brain injury but provide limited insights to other components of traumatic brain injury, such as axonal injury. Last, due to ethical and technical reasons, the long-term outcome, for example, 6 months after the insult, could not have been assessed.

## 5 Conclusion

In this exploratory study using a model with cardiovascular healthy pigs, targeted hyperoxemia following ASDH and HS significantly improved MGCS while not significantly affecting survival. Targeted hyperoxemia had no significant beneficial nor deleterious effects. More studies with larger case numbers need to confirm the findings of the present study to address the unmet clinical need to improve the treatment of patients with TBI.

## Data availability statement

The original contributions presented in this study are included in the article/**Supplementary Material**. Further inquiries can be directed to the corresponding author.

## Ethics statement

The animal study was reviewed and approved by Local Animal Care Committee of Ulm University and the Federal Authorities for Animal Research.

## Author contributions

Conceptualization. PR. Methodology. TD, AH, TK, RM, SM, HG, EC, PR, DM. Validation. PR, DM. Formal Analysis. TD, FM, AH, EM, MB, PR, DM. Investigation. TD, AH, EM, MB, MG, TK, RM, SM, HG, EC, PR, DM. Resources. PR. Data curation. PR, DM. Writing – Original Draft. PR, DM. Writing – Review & Editing. All authors. Visualization. DM. Supervision. EC, PR. Project Administration. AH, PR, DM. Funding Acquisition. PR. All authors contributed to the article and approved the submitted version.

## Supplementary material

Supplemental Figure 1 Results of the metabolic situation at the site of ASDH (ipsilateral) and the opposing hemisphere (contralateral). Measurements were recorded before (pre) and after 2 hrs of hemorrhagic shock and acute subdural hematoma (post) as well as 24 hrs and 48 hrs after resuscitation. n = 3/5 before shock, 5/5 after shock, 4/5 at 24 hrs, and 1/4 at 48 hrs per group for normoxemia (blue, control group, C) and targeted hyperoxemia (red, H), respectively. **(A)** Glucose ipsilateral, **(B)** glucose contralateral, **(C)** glutamate ipsilateral, **(D)** glutamate contralateral, **(E)** pyruvate ipsilateral, **(F)** pyruvate contralateral, **(G)** lactate ipsilateral, and **(H)** lactate contralateral. Mann-Whitney U test. Box plots report median, interquartile range, minimum, and maximum.
